# Multiple Facets of Autophagy and the Emerging Role of Alkylphosphocholines as Autophagy Modulators

**DOI:** 10.3389/fphar.2020.00547

**Published:** 2020-04-29

**Authors:** Ferda Kaleağasıoğlu, Doaa M. Ali, Martin R. Berger

**Affiliations:** ^1^Department of Pharmacology, Faculty of Medicine, Near East University, Mersin, Turkey; ^2^Toxicology and Chemotherapy Unit, German Cancer Research Center (DKFZ), Heidelberg, Germany; ^3^Department of Pharmacology and Experimental Therapeutics, Medical Research Institute, Alexandria University, Alexandria, Egypt

**Keywords:** types of autophagy, autophagy as drug target, alkylphosphocholines, Akt/mTOR pathway, miltefosine/perifosine/erufosine

## Abstract

Autophagy is a highly conserved multistep process and functions as passage for degrading and recycling protein aggregates and defective organelles in eukaryotic cells. Based on the nature of these materials, their size and degradation rate, four types of autophagy have been described, *i.e.* chaperone mediated autophagy, microautophagy, macroautophagy, and selective autophagy. One of the major regulators of this process is mTOR, which inhibits the downstream pathway of autophagy following the activation of its complex 1 (mTORC1). Alkylphosphocholine (APC) derivatives represent a novel class of antineoplastic agents that inhibit the serine–threonine kinase Akt (*i.e.* protein kinase B), which mediates cell survival and cause cell cycle arrest. They induce autophagy through inhibition of the Akt/mTOR cascade. They interfere with phospholipid turnover and thus modify signaling chains, which start from the cell membrane and modulate PI3K/Akt/mTOR, Ras-Raf-MAPK/ERK and SAPK/JNK pathways. APCs include miltefosine, perifosine, and erufosine, which represent the first-, second- and third generation of this class, respectively. In a high fraction of human cancers, constitutively active oncoprotein Akt1 suppresses autophagy *in vitro* and *in vivo*. mTOR is a down-stream target for Akt, the activation of which suppresses autophagy. However, treatment with APC derivatives will lead to dephosphorylation (hence deactivation) of mTOR and thus induces autophagy. Autophagy is a double-edged sword and may result in chemotherapeutic resistance as well as cancer cell death when apoptotic pathways are inactive. APCs display differential autophagy induction capabilities in different cancer cell types. Therefore, autophagy-dependent cellular responses need to be well understood in order to improve the chemotherapeutic outcome.

## Introduction

Autophagy is derived from a Greek word (αυτοϕαγϵἷν) meaning ‘self-eating’ and is a bulk degradation process, which includes the lysosomal-dependent degradation and recycling of components of eukaryotic cells. It has essential roles in keeping the cellular homeostasis and functions in cellular differentiation, control of cellular growth, cell defense, and promotes tissue remodeling and acclimatization. Autophagy either can have a protective function for cell survival or promote cell death. Alkylphosphocholines (APCs) are phospholipid-derived agents that cause changes in cell signaling by enriching in cell membranes including the lipid rafts. This physicochemical property and the resultant changes are basis for their anticancer, antiprotozoal, antibacterial, and antiviral activities. They induce autophagy as part of their mechanism of action. This review summarizes the current knowledge on alkylphosphocholines (APCs) regarding their influence on autophagy. It covers the following topics: Types of autophagy, role of autophagy in cancer, autophagy as a therapeutic target, APCs (miltefosine, perifosine, and erufosine) in general, as modulators of autophagy, and conclusion.

## Types of Autophagy

Four types of autophagy have been recognized based on the nature of their cargo, their cargo size, and degradation rate, *i.e.* chaperone mediated autophagy (CMA), microautophagy, macroautophagy, and selective autophagy (Hayashi-Nishino et al., 2009; Bejarano and Cuervo, 2010; Li et al., 2012; Lippai and Szatmari, 2017; Bednarczyk et al., 2018). The lysosomal degradation of damaged proteins is common to all the previously mentioned types of autophagy, but the mechanism of delivering the substrate to the lysosome varies among the different types (Bednarczyk et al., 2018). The following part gives a short description of these four types.

### Chaperone Mediated Autophagy

**Figure 1:**
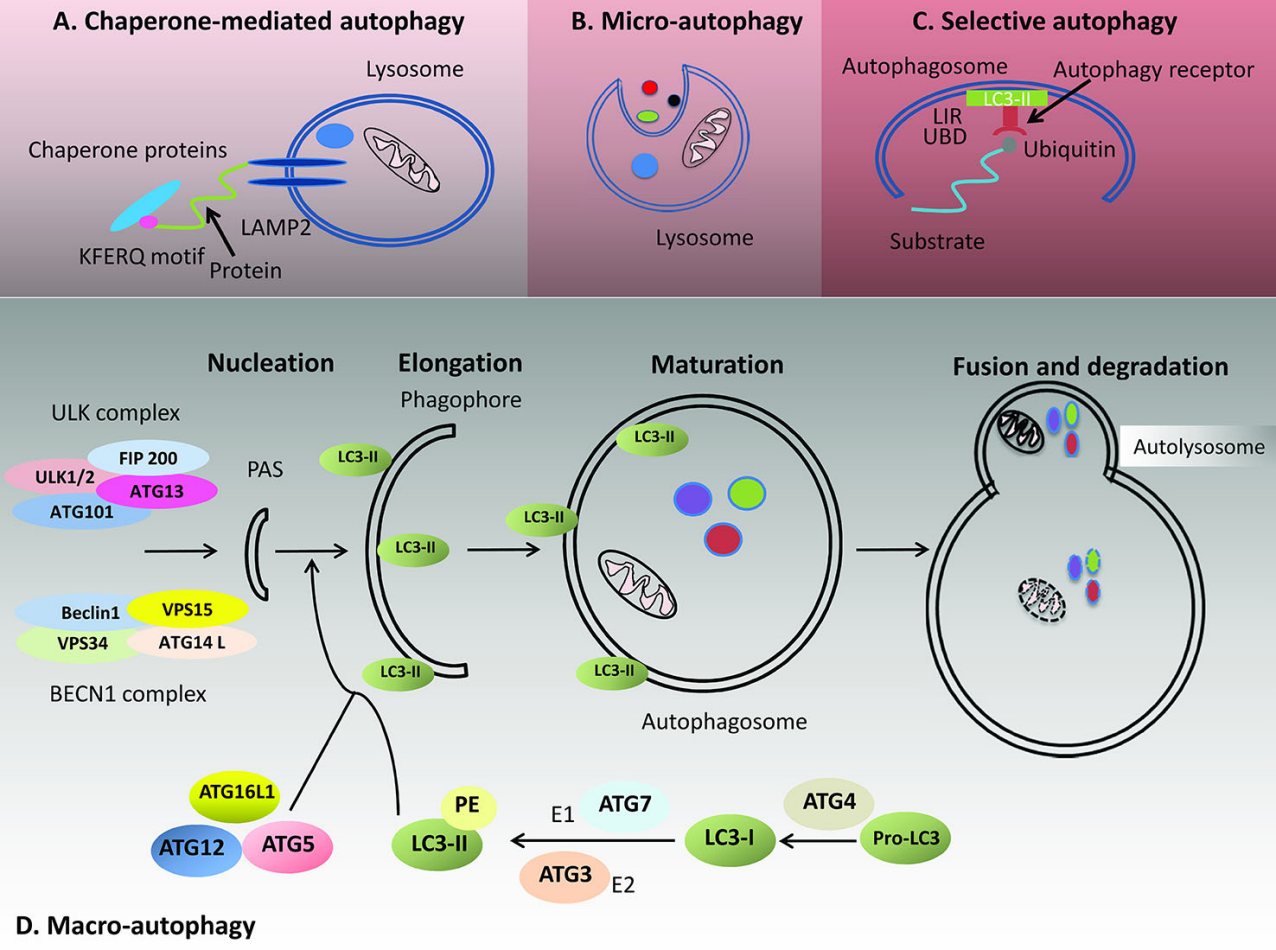
Types of autophagy: The four types of autophagy include chaperone mediated autophagy, microautophagy, selective autophagy, and macroautophagy. **(A)** Chaperone-mediated autophagy involves the recognition of a KFERQ penta-peptide motif in substrate proteins by corresponding chaperone proteins. The substrate is then transferred to the lysosomal lumen after binding to the LAMP protein. **(B)** Microautophagy is the process of sequestering minute parts of the cytoplasm and their engulfment by lysosomal invagination. **(C)** During selective autophagy, the respective cargo (*e.g.* invading pathogens, damaged mitochondria, or others) is specifically bound by autophagy receptors. The autophagy receptor has the ability to bind LC3 proteins through its LC3 interacting region (LIR) on the autophagosome beside binding molecular determinants, such as unfolded regions of a protein or conjugated ubiquitin (Ub) through its ubiquitin binding domain (UBD). **(D)** Macroautophagy consists of several steps of nucleation, elongation, maturation, and finally fusion and degradation. The process starts by the association of the ULK1 and BECN1 complexes that form the basis for recruiting other autophagy-related (ATG) proteins as well as the lipidated form of LC3 (LC3-II, *i.e.* LC3-I linked to phosphatidyl-ethanolamine). The ULK1 complex consists of the serine/threonine kinase UNC-51-like autophagy activating kinase (ULK1), focal adhesion kinase family interacting protein of 200 kDa (FIP200), ATG13, and ATG101. The PI3 kinase III nucleation complex (BECN1 complex) consists of Beclin-1, class III phosphoinositide 3-kinase [PI3K-III; also termed vacuolar protein sorting 34 (VPS34)] and its regulatory subunit VPS15. LC3-I protein is formed from its precursor protein, pro-LC3 with the contribution of ATG4. After attaching phosphatidyl-ethanolamine (PE) to LC3-I by ATG7 and ATG3, the lipophilic form (LC3-II) is created. The closed autophagosome fuses with a lysosome to form the autolysosome, where the proteins undergo degradation by different lysosomal enzymes.

Chaperone proteins recognize substrate proteins for chaperone mediated autophagy (CMA, see **Figure 1A**) by the penta-peptide motif KFERQ (*i.e.* K: lysine–F: phenylalanine–E: glutamic or aspartic acid–R: arginine–Q: glutamine) (Jackson and Hewitt, 2016; Bednarczyk et al., 2018). Together with their chaperones, these proteins will be transported to lysosomes for breakdown in a receptor dependent manner (Bejarano and Cuervo, 2010). The native state of a substrate protein usually hides the recognition motif within the protein core, but it comes to be accessible by its respective chaperone regardless of its location within the protein. Examples for chaperones include heat shock cognate protein of 70 kD (Hsp70) and cochaperones as Hsp90, Hsp40, and Bcl-2-associated athanogene 1 (Bag-1). The latter will unfold the proteins before substrate–chaperone interactions can occur, eventually even without direct interaction. Then, the substrate is transferred to the lysosomal lumen after binding to the cytosolic tail of lysosome-associated membrane protein type 2A (LAMP-2A), which multimerizes to that purpose (Agarraberes and Dice, 2001; Bandyopadhyay et al., 2008; Bejarano and Cuervo, 2010; Jackson and Hewitt, 2016). CMA activity is directly proportional to the level of LAMP2A in the lysosomal membrane. CMA consists of four stages: recognition of the substrate, substrate binding, substrate translocation, which is an ATP dependent step, and finally substrate hydrolysis within the lysosome by proteolytic enzymes (Cuervo and Wong, 2014). Hsp90 maintains the stability of LAMP2A receptor during multimerization (Cuervo and Wong, 2014). Starvation of more than 10 h, oxidative stress, and exposure to toxic compounds will induce CMA. Under such conditions, the level of LAMP2A increases to meet the requirement of increasing CMA, and this occurs through degradation of the LAMP2A complex and transferal of its constituent proteins to the lysosomal membrane (Bednarczyk et al., 2018). The levels of LAMP2A protein are clearly organized by degradation at the lysosomal membrane, distribution between this structure and its lumen, or by *de novo* synthesis (Kaushik and Cuervo, 2006). The interaction of substrate and chaperone to form a complex is considered the rate-limiting step of the CMA process (Kaushik and Cuervo, 2006). However, the by age abridged stability of LAMP2A reduces CMA, which initially can be compensated by increased lysosome numbers (Kaushik and Cuervo, 2006; Bednarczyk et al., 2018). Reduced CMA activity as age-related effect results mainly in deficient binding and uptake of substrates into the lysosomal membrane, while kinetics of degradation is comparable to younger ages. The reduced CMA activity may have a role in the accumulation of altered products observed with aging (Kaushik and Cuervo, 2006).

### Microautophagy

Microautophagy denotes the process of sequestering tiny parts of the cytoplasm and their subsequent engulfment through lysosomal invagination as shown in **Figure 1B** (Mijaljica et al., 2014).

Five phases of microautophagy have been identified: the first phase is the microautophagic invagination and formation of autophagic tubes, where the normal membrane bulges by lateral seclusion of lipids and local segregation of large transmembrane proteins toward the surface of lysosomes or vacuoles, and by an ATP dependent process then forms an autophagic tube. The second phase is vesicle formation, which is the equivalent of autophagosome formation in macroautophagy. It occurs because of the lateral organizing mechanism, where autophagic tubes invaginate because high-density lipids combine with low-density proteins. Vesicle expansion is the third step, which is characterized by the hanging of a prevesicular structure that dynamically moves back and forth in the lysosomal/vacuolar lumen. The formed vesicle does not contain proteins but lipids of high density because of the mechanism of lateral sorting. Finally, vesicle scission occurs because of the dynamic trend, where one or two vesicles bud into the lumen of the lysosome and move freely at high speed. Vesicle degradation happens because of the effect of some hydrolases that break down the freely moving vesicles. Recycling of the nutrients is done by a permease like action of ATG22p (Li et al., 2012).

### Selective Autophagy

As compared to macroautophagy, which is considered nonselective, selective autophagy (**Figure 1C**) ensures recognition and elimination of specific cytosolic cargoes. Selective autophagy is specific for its substrates *e.g.*, for damaged mitochondria (mitophagy), aggregated lipids (lipophagy), invading pathogens (xenophagy), or excess peroxisomes (pexophagy); these specific cargoes undergo degradation after being identified by autophagy receptors and are encircled into a double-membrane vesicle, the autophagosome, and transported to the lysosome for further breakdown (Rogov et al., 2014). The specificity is determined by identification of selective autophagy receptors (Rogov et al., 2014), which have the ability to bind LC3/GABARAP proteins on the forming autophagosome in addition to binding molecular elements, such as unfolded regions of a protein or conjugated ubiquitin (Ub). Then, through self-oligomerization, they contribute to the association of specific platforms on which autophagosomes form. Among the autophagy receptors are p62/sequestosome 1 (p62/SQSTM1), optineurin (OPTN), neighbor of BRCA1 (NBR1), and nuclear dot protein 52 kDa (NDP52). All of them possess a ubiqitin-binding domain (UBD) and LC3-interacting regions (LIRs) (Rogov et al., 2014; Stolz et al., 2014; Deng et al., 2017; Bednarczyk et al., 2018).

### Macroautophagy

Macroautophagy (see **Figure 1D**, will be referred later as autophagy) involves the formation of isolation membranes (IMs), which extend as they ingest parts of the cytoplasm and organelles to produce autophagosomes (Hamasaki et al., 2013). This whole process is nonselective (Bednarczyk et al., 2018). Autophagosomes are double membrane structures, which function in delivering cargos to lysosomes or endosomes (Eskelinen, 2008). Autophagy is a complex mechanism with the involvement of several autophagy related proteins. Autophagy related proteins (ATG) responsible of the autophagy process were essentially discovered from yeast genome (Klionsky et al., 2003; Eskelinen, 2008). The source of the autophagosomal membrane is uncertain, as it is discussed to originate from endoplasmic reticulum (ER), mitochondria, or plasma membranes (Tooze and Yoshimori, 2010; Hamasaki et al., 2013; Nascimbeni et al., 2017; Wei et al., 2018). Hailey and coworkers found that the external membrane of the mitochondria adds to autophagosome production in fasting cells (Hailey et al., 2010). Both the endoplasmic reticulum (ER) and the early autophagic structures of IMs are interconnected. Electron tomography showed that the ER–IM complex is a subdomain of the ER that forms a frame surrounding the IM (Hayashi-Nishino et al., 2009). Other studies suggested’ that autophagosomes form at the ER–mitochondria contact site in mammalian cells (Hamasaki et al., 2013), or that the plasma membrane serves as a reservoir and participates openly in the development of positive autophagosome precursors during periods of increased autophagosome formation (Ravikumar et al., 2010). Furthermore, studying the intracellular dynamics of ATG9 in yeast showed that the Golgi apparatus derived ATG9 vesicles incorporate into the autophagosomal outer membrane at the initial stages of autophagosome generation (Yamamoto et al., 2012). Recycling endosomes represent membrane platforms that contribute to the formation of phagophores (Puri et al., 2018).

#### Mechanism and Regulation of Autophagy

Autophagy constitutes of several sequential steps, namely initiation or nucleation, phagophore elongation, autophagosome maturation, autophagosome fusion with the lysosome, and proteolytic degradation of the contents (Kardideh et al., 2019). The initial step usually begins with the association of the initiation complex (ULK1 complex). This complex consists of ULK1 (UNC-51-like autophagy activating kinase, a serine/threonine kinase), FIP200 (focal adhesion kinase family interacting protein of 200 kDa), ATG13 and ATG101, together with the nucleation complex (BECN1 complex) consisting of Beclin-1, class III phosphoinositide 3-kinase [PI3K-III or vacuolar protein sorting 34 (VPS34)] and its regulatory subunit VPS15. The latter represent the platform for recruiting other ATG proteins and for elongating the phagophore membrane (Lippai and Szatmari, 2017; Bednarczyk et al., 2018).

The three proteins VPS34, VPS15, and BECN1 make up the core, induce the elevated phosphatidylinositol 3-phosphate (PI3P) level of autophagic membranes, and can form, together with a fourth subunit, two different complexes. When this subunit is the UV radiation resistance associated (UVRAG) protein, the complex plays an essential role in endosomal maturation, but when the complex harbors ATG14L, it is required for autophagy. Engaging the BECN1 core complex to the phagophore assembly site (PAS) requires the function of ATG14L. Lipidation of ATG8/LC3 with phosphatidyl-ethanolamine (PE) is a crucial step in elongating the phagophore. Two ubiquitin-like conjugation systems act on this, namely: ATG7 with its E1 enzyme-like protein stimulating activity and ATG10 with its E2 conjugation enzyme like function. Their activities result into covalent bonding between ATG12 and ATG5. A multimeric complex, made of ATG5-ATG12 and ATG16L1, functions as a ubiquitin ligase like enzyme and facilitates binding of ATG8/LC3 to PE. Prerequisite to this step, LC3-I protein is formed by processing its precursor protein pro-LC3 with the protease ATG4. Then ATG7 and ATG3 activate ATG8 by E1 and E2 like enzymatic actions, and as a result, the lipophilic form (LC3-II) is created and bound to both membrane leaflets of the phagophore (Lippai and Szatmari, 2017). LC3-II can be considered as the best marker of autophagy, as its concentration is directly proportional to the number of autophagosomes formed (Burada et al., 2015) (Bednarczyk et al., 2018). All ATGs, with the exception of ATG8/LC3, dissociate from the membrane before closure and are recycled. Recycling of ATG8/LC3 occurs after closure of the autophagosome with the help of ATG4, while lysosomal enzymes in the autophagosome lumen cleave the proteins attached to the internal membrane (Lippai and Szatmari, 2017). Autophagosomes bind to late endosomes and lysosomes to form the autolysosomes and proceed for degradation (Lippai and Szatmari, 2017). LAMP proteins regulate the fusion and prevent degradation of the lysosomal membrane (Huynh et al., 2007; Lippai and Szatmari, 2017). The merger of the autophagosome with the lysosome to result in the autolysosome is facilitated by either the soluble NSF (N-ethylmaleimide sensitive factor) attachment protein (SNAP) receptor (SNARE) protein complex, or UVRAG, but many players can be involved in this process including cytoskeleton constituents and associated motor proteins, tethering factors, phospholipids, and specific SNARE complexes. This step is finished by degrading the interior autophagosomal membrane by lysosomal enzymes (Bednarczyk et al., 2018; Yu et al., 2018). **Figure 1D** shows the follow-up of steps described for macroautophagy.

Autophagy as a process is highly conserved and firmly controlled in mammals. The most important physiological regulator of autophagy is the availability of nutrients and amino acids. Other regulators include mTOR, especially its complex 1 (mTORC1), and inhibition of mTORC1 results in autophagy induction. In addition, starvation and amino acid depletion result into autophagy induction. This usually happens through activating adenosine monophosphate-activated protein kinase (AMPK), which transfers a phosphate group to ULK1, or by impeding mTORC1 activity, or through inhibitory phosphorylation of nonautophagic BECN1 complexes. Other autophagy regulators depend on an increase in cytosolic calcium, inhibition of inositol triphosphate or starvation induced autophagy (Petiot et al., 2000; Eskelinen, 2008; Jewell et al., 2013; Lippai and Szatmari, 2017). Furthermore, oxidative stress (Filomeni et al., 2015), DNA damage (Gomes et al., 2017) as well as hypoxia (Fang et al., 2015) can all induce autophagy. Activation of class I PI3-kinases inhibits autophagy, while class III PI3-kinase activity is required for autophagosome formation.

Autophagy breaks macromolecules and then provides nutrients and functions as a survival mechanism during short-term starvation (Eskelinen, 2008; Lippai and Szatmari, 2017). It eliminates damaged proteins and organelles and helps in the organelle turnover. It fights against attacking pathogens and in general preserves the cellular homeostasis and balance (Eskelinen, 2008; Lippai and Szatmari, 2017).

Autophagy also results into type II programmed cell death and is related to apoptosis in several ways. The tumor suppressor death-associated protein kinase (DAPk) may contribute to the signaling pathway linking autophagy to cell death. Certain types of cell death depend on autophagy proteins for the execution of cell death. Autophagy can protect cells from the apoptotic fate by providing nutrients, especially under conditions of starvation. The regulation of both processes is related to the pro-survival protein Bcl-2. Bcl-2 binds to Beclin 1 and prevents its interaction with VPS34. Thus, it inhibits the Beclin 1 dependent autophagy and maintains autophagy at levels harmonious with cell survival, rather than cell death (Eskelinen, 2008; Bednarczyk et al., 2018).

Different human diseases are associated with deregulation of autophagy, including neurodegenerative diseases and proteinopathies, lysosomal disorders, many cardiovascular diseases, cancer, diabetes and immune disorders. Infections and pathogens control autophagy based on their needs to assure their persistence in host cells (Lippai and Szatmari, 2017).

#### mTOR Regulation of Autophagy

Autophagy is controlled by a negative feedback mechanism of the mTOR pathway and modulators of this pathway have an impact on and can regulate autophagy (Jung et al., 2010; Kim and Guan, 2015). In normal cellular states and when amino acids are abundant, mTORC1 binds to the ULK complex by interaction of its component RAPTOR with ULK1. Then, mTOR transfers phosphate groups to ULK1 (specifically at its S757 and S637 residues) and ATG13 and thus hinders the ULK1 kinase activity. Under fasting conditions, the mTOR regulatory pathway is inhibited, and this leads to induction of autophagy because mTORC1 separates from the ULK complex and therefore its inhibitory control on ULK1 is gone, which allows autophagy to proceed (Rabanal-Ruiz et al., 2017). LKB1, AMPK, TSC1/TSC2 complex, and PTEN in mTOR signaling pathways can induce autophagy, while Akt and Rheb have an inhibitory effect. mTOR is inhibited at the beginning of autophagy and becomes activated later on, due to the release of breakdown molecules in the cytoplasm, which result into inhibition of the whole process. Enlarged mTOR activity then impedes autophagy and induces the formation of proto-lysosomal extensions (LAMP1+, LC3−) from autolysosomes (LAMP1+, LC3+). Finally, these proto-lysosomal extensions separate from the autolysosome and advance into functional lysosomes. Impediment of mTOR or its (auto-) lysosomal activity precludes autophagic lysosome restoration (Kapoor et al., 2014).

## Role of Autophagy In Cancer

In carcinogenesis, the role of autophagy is controversial with many conflicting reports in the literature. Autophagy can either impede or favor cancer development and progression, which depends on the wild-type or transformed state of the cell, the underlying genetic lesion(s), the tumor type and stage, as well as the tumor microenvironment (Galluzzi et al., 2015; New et al., 2017; Bednarczyk et al., 2018; Kardideh et al., 2019).

Under normal conditions, autophagy acts as a protector against cancer development. In contrast, during stress situations, autophagy helps cells to adapt against hypoxia and nutrient deficiency and can save cancer cells from death (Galluzzi et al., 2015; New et al., 2017; Bednarczyk et al., 2018). Fighting mutagenic effects (*e.g.* DNA damage or instability of the genome) occurring from accumulation of reactive oxygen species (ROS) and degrading of oncogenic proteins are among the protective functions that autophagy can exert to inhibit cancer induction (Galluzzi et al., 2015; New et al., 2017; Bednarczyk et al., 2018). A reduced level of autophagy will hinder the ability of the cells to eliminate impaired proteins and damaged organelles and hence begin to mount up cytotoxic components that can cause damage to DNA and initiate carcinogenesis (Bednarczyk et al., 2018). Autophagy can suppress tumorigenesis through cell cycle and cell death regulation in conjunction with the ubiquitin–proteasome system (UPS), which can modify some key cell cycle components of CDK-Cyclin complexes (Kardideh et al., 2019).

Autophagy is required for immune activation as it plays a role in antigen presentation to T lymphocytes. It helps the maturation of some innate immune cells when activated and contributes to their antitumor activity. It can play a role in combatting cancer through activation of the immune system (Janji et al., 2018). Nevertheless, hypoxia-induced autophagy may result in the activation of immune escape mechanisms in the tumor microenvironment (Janji et al., 2018). Regarding the anticancer effects, some autophagic genes and inducers are mutated or deleted in certain cancers, as p53 and PTEN (phosphatase and tensin homolog), which are the most frequently altered tumor suppressor genes, and Beclin-1 (BECN1), which is deleted in breast and ovarian cancers (Bednarczyk et al., 2018).

Regarding the tumor supportive roles, autophagy provides tumor cells access to nutrients that are crucial to their metabolism, promotes DNA repair, reduces mitochondrial disorders, and increases drug resistance (Burada et al., 2015; Bednarczyk et al., 2018). Autophagy also helps tumor cells to resist stress and apoptotic signals; it provides cells with energy through increasing ATP concentrations that favor cell survival during hypoxia and starvation. Autophagy is linked in late cancer to poor prognosis and invasiveness (Galluzzi et al., 2015; New et al., 2017) and favors tumor growth through rendering cells more resistant to apoptotic signals and stress stimuli as well as resistance to therapy induced cell death. Furthermore, autophagy maintains the neoplastic stem cell compartment and epithelial-to-mesenchymal transition (EMT) (Galluzzi et al., 2015).

In metastasis, autophagy can also play a dual role, based on the stage. In early stages, autophagy may stimulate inflammatory responses and limit tumor necrosis. Further, autophagy may limit the development of dormant cancer cells into micrometastases, as well as prevent oncogene induced senescence. However, in advanced stages, autophagy tends to increase the life span of circulating metastatic cells, which lack an extracellular matrix, by inducing dormancy in the new environment until favorable conditions occur (Burada et al., 2015).

In this context, the autophagosome marker LC3B showed moderate to high expression in solid cancers, as breast cancer and melanomas, and its expression is linked to cell growth, invasion and metastasis, high tumor grade, and poor prognosis (Lazova et al., 2012).

To summarize, autophagy protects against malignant transformation under normal conditions by maintaining the cellular homeostasis, but increases tumor progression and invasiveness after tumor establishment (Galluzzi et al., 2015; New et al., 2017). The different roles of autophagy in cancer are shown in **Table 1**.

**Table 1:** The dual role of autophagy in cancer^a)^.

Onco-stimulatory roles	Onco-suppressive roles
Support of cancer stem cells	Clearance of intracellular pathogens
Providing cells with an alternative energy source	Favoring the genomic stability
Inhibition of cell death	Reduction of DNA damage and reactive oxygen species
Reduction of cellular sensitivity to stress stimuli	Reduction of inflammation
Maintaining the dormancy state of tumor cells until favorable conditions are met	Stimulation of autophagic cell death
Promotion of drug resistance	Removal of damaged organelles and proteins

^a)^modified from (Burada et al., 2015; Bednarczyk et al., 2018).

### Aspects of Autophagy in Disease Prevention

The physiologic functions of selective autophagy recently have been perceived as potentially preventive measures. Basis for this concept is *e.g.* the observation that aging mammalian cells accumulate dysfunctional mitochondria, which can be eliminated by selective autophagy (Hansen et al., 2018). Mitophagy, *i.e.* the elimination of aged mitochondria is therefore a concept of antiaging strategies as well as for diseases that are characterized by a pathophysiology, which is related to mitochondrial disorders, including heart disease, retinopathy, and a series of neurodegenerative sicknesses as *e.g.* Alzheimer’s disease. Tolerable induction of mitophagy could thus pave the way to neuroprotection and healthy longevity (Fang et al., 2014; Lou et al., 2020). Calorie restriction, low insulin/IGF1 levels, and the intake of NAD+ precursors are examples of a strategy aiming to increase the clearance of damaged mitochondria. NAD+ is a cofactor of the NAD-dependent deacetylase sirtuin-1, which can induce mitophagy (Lee et al., 2008). Sirtuin activating compounds include resveratrol, which together with NAD+ precursors (tryptophan, nicotinic acid, nicotinamide, nicotinamide mononucleotide, and nicotinamide riboside) could be basis of a dietary approach to healthy longevity (Bonkowski and Sinclair, 2016).

## Autophagy as Therapeutic Target

Autophagy maintains the intracellular metabolic homeostasis, and its dysfunction is associated with numerous diseases including cancer, neurodegeneration, cardiac ischemia, metabolic dysfunctions, infections, autoimmune and pulmonary disorders. Aging and associated atherosclerotic cardiovascular diseases may be slowed down by increasing autophagic flux through calorie restriction, fasting, exercise, and nutritional support such as with spermidine-rich food (Maiuri and Kroemer, 2019). Autophagy is also being considered as a “druggable” process due to its fine regulation by diverse signaling pathways, hence involvement of multiple targets (Galluzzi et al., 2017; Morel et al., 2017). Whether pharmacological modulation of autophagy may be advantageous over life-style interventions remains an open question (Maiuri and Kroemer, 2019).

Autophagy as a therapeutic target in cancer is a double-edged sword, which can both enhance neoplastic growth or suppress cancer cell survival. Cancer cells can adapt to hyponutrient conditions and protect themselves against cancer chemotherapeutics by increasing autophagic flux (Yoshida, 2017). At the same time, induction of autophagy enables antigen cross-presentation, which stimulates antitumor immune response and may protect against relapses (Maiuri and Kroemer, 2019). On the other hand, autophagy contributes to multidrug resistance (MDR) development. Autophagy inhibitors or silencing of ATGs by microRNAs can thus sensitize cancer cells to chemotherapy, enable the use of lower dosage, and reduce adverse effects. However, autophagy inducers may also reverse MDR and sensitize apoptosis-resistant MDR cells to cancer chemotherapeutics (Li et al., 2017).

Deregulated autophagy can be modulated by either inhibiting or inducing identified targets in relevant pathways. Preclinical data followed by clinical trials demonstrate that some old drugs with already well-known safety profiles may be beneficial as autophagy modulators in certain diseases. Among these “conventional” drugs, metformin, the drug of choice in type 2 diabetes mellitus, is also being recognized as an antiaging agent because of its autophagy enhancing effect. In addition, antimalarial drugs have gained attention as autophagy inhibitors, including chloroquine (CQ) and hydroxychloroquine (HCQ). They are the mostly investigated drugs for this purpose in clinical studies, especially in cancer. Other autophagy inducers like mTOR inhibitors, BH3 mimetics, the receptor tyrosine kinase inhibitor sorafenib, the proteasome inhibitor bortezomib, the antigout agent colchicine, the nonreducing disaccharide trehalose, and 2-deoxyglucose have entered clinical phase trials against cancer and neurodegenerative diseases.

**Figure 2:**
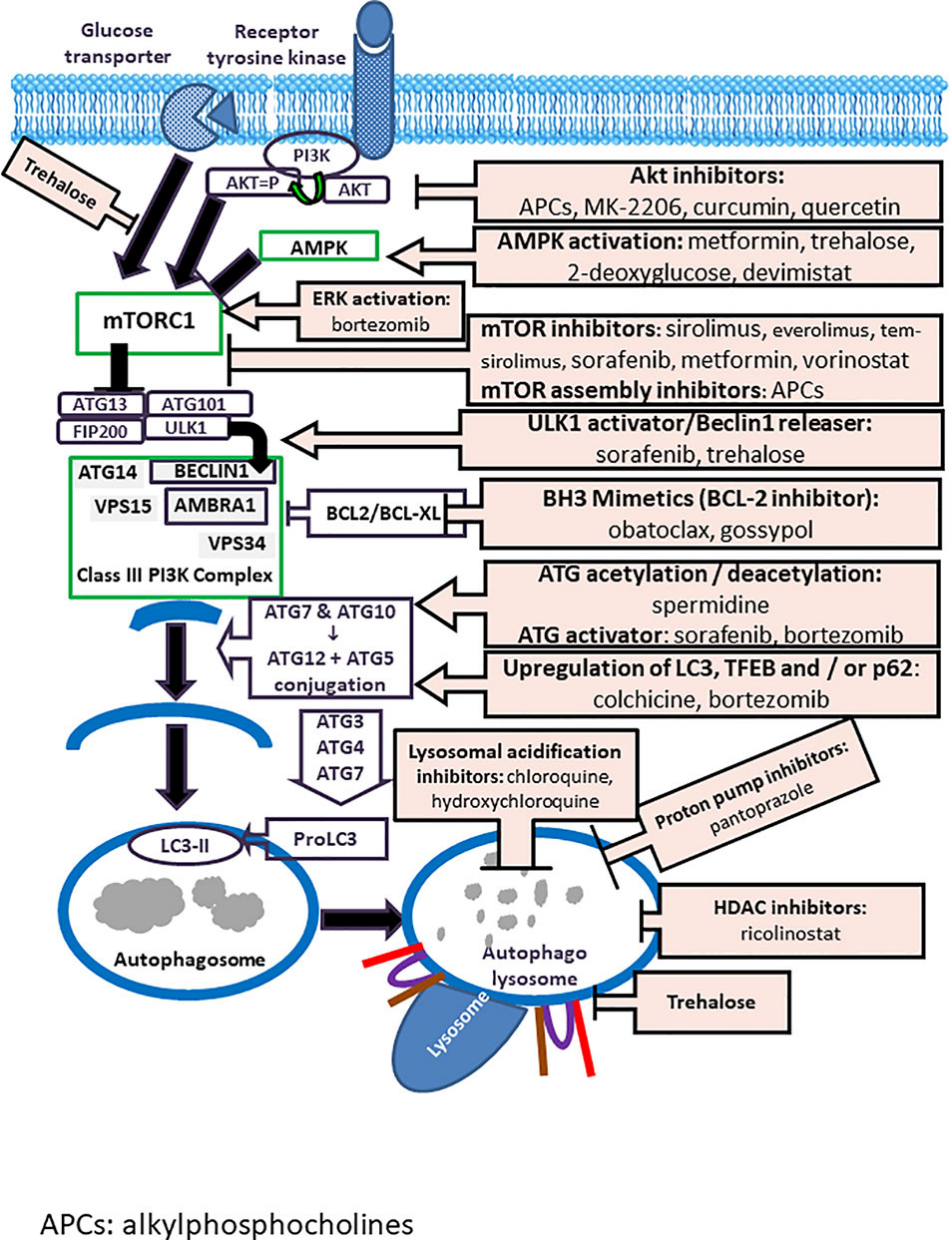
Modulators of autophagy Inhibitors of autophagy include Akt-inhibitors (alkylphosphocholines, MK-2206, curcumin, quercetin); AMPK activators (metformin, trehalose, 2-deoxyglucose, devimistat); ERK activators (bortezomib); mTOR-inhibitors (sirolimus, temsirolimus, sorafenib, metformin, vorinostat); mTOR-assembly inhibitors (alkylphosphocholines); ULK1 activators/Beclin releasers (sorafenib, trehalose); BH3-mimetics/BCL-2 inhibitors (obatoclax, gossypol); acetylators/deacetylators/activators of autophagy related (ATG) proteins (spermidine, sorafenib, bortezomib); upregulators of LC3; TFEB/p62 (colchicine, bortezomib); inhibitors of lysosomal acidification (chloroquine, hydroxychloroquine); proton pump inhibitors (pantoprazol), and inhibitors of the fusion of lysosomes with autophagosomes (trehalose).

Many novel autophagy modulators were evaluated in preclinical trials, but only some of these molecules have succeeded to pass the clinical development phase, and most of the phase trials were based on repurposing of old drugs. Macroautophagy modulators, which have entered phase I–III trials and their mechanisms of action are illustrated in **Figure 2**. The key inhibitor of autophagy, mTORC, can be inhibited by AKT-inhibitors (alkylphosphocholines, see below) and mTOR-inhibitors (sirolimus, everolimus, temsirolimus, sorafenib, metformin, and vorinostat), and by adenosine monophosphate-activated protein kinase (AMPK) activation (devimistat, metformin and trehalose). The other two autophagy targets in the downstream pathway are ULK1 and BECLIN1, which can be activated by ULK1 activator/BECLIN1 releasers such as sorafenib, trehalose, gossypol and BH3 mimetics (BCL-2 inhibitors like obatoclax). Sorafenib and bortezomib can also induce ATG activation. Autophagic flux can be enhanced by endoplasmic reticulum stress and related unfolded protein response (UPR) signaling (alkylphosphocholines, sorafenib, 2-deoxyglucose), inhibition of glycolysis (2-deoxyglucose) and blockade of the cargo receptor p62/sequestome1 (p62/SQSTM1) degradation (bortezomib). The histone deacetylase 6 (HDAC6) inhibitor ricolinostat, lysosomal acidification inhibitors (CQ, HCQ), proton pump inhibitors (pantoprazole) and the autophagosome–lysosome fusion inhibitor trehalose can inhibit autophagosome maturation and autophagolysosomal degradation. The detailed mechanisms of actions of these autophagy modulators are given in **Table 2**.

**Table 2:** Autophagy modulators in clinical trials and their mechanisms of action.

Modulator	Modulation	Mechanism of Action	Reference
HCQ and CQ	Inhibition	Inhibition of lysosomal acidification and thus the blockade of the terminal step of autophagic substrate degradation	(Perez-Hernandez et al., 2019)
Rapamycin (sirolimus) Everolimus Temsirolimus	Activation	mTOR inhibition.	(Mandrioli et al., 2018)
Pantoprazole	Inhibition	Inhibition of H+/K + ATPase proton pumps in membranes of intracellular endosomes and increase of endosomal pH, resulting in inhibition of autophagosome maturation.	(Marino et al., 2010)
Metformin	Activation	Activation of AMPK, a sensor of cellular energy levels (increased AMP/ATP ratio) in the cell	(Novita et al., 2019)
Ricolinostat (ACY-1215)	Inhibition	Selective inhibition of HDAC6, which mediates trafficking of ubiquitinated misfolded proteins to the aggresome/autophagy pathway.	(Vogl et al., 2017)
Vorinostat (suberoylanilide hydroxamic acid; SAHA)	Activation	Pan-HDAC inhibitor; inhibition of mTOR, which results in the dephosphorylation, and thus activation, of the autophagic protein kinase ULK1 and increases LC3 expression.	(Gammoh et al., 2012)
Devimistat (CPI-613)	Inhibition	Inhibition of pyruvate dehydrogenase and ketoglutarate dehydrogenase of TCA cycle, thus impairment of pancreatic cell mitochondrial metabolism.	(Philip et al., 2019)
Spermidine	Activation	Suppression of acetyltransferase activity of EP300, and inducing the acetylation or deacetylation of autophagy-related genes (Atgs).	(Yang et al., 2017)
Bortezomib	Inhibition; activation	Induction of ERK phosphorylation to suppress cathepsin B, thus inhibition of the catalytic process of autophagy; blockade of p62 degradation. Induction of autophagy through endoplasmic reticulum stress.	(Kao et al., 2014) (Li et al., 2018b)
Sorafenib	Activation	Activation of HP1-STAT3-Mcl-1-Beclin1 pathway and releasing Beclin1 from Mcl-1; mTORC1 inhibition; activation of IRE1 signaling pathway of ER stress, thus reduction of ER stress-induced cell death; activation of AMPK.	(Sun et al., 2017)
Colchicine	Activation	Upregulation of proteins involved in autophagy, including the master regulator transcription factor EB (TFEB), the TFEB regulated adaptor protein SQSTM1/p62 and autophagy player microtubule-associated protein 1A/1B-light chain 3 (LC3)	(Mandrioli et al., 2019)
^188^Re-liposome	Inhibition	Microtubule-associated protein 1 light chain 3B (LC3) and lysosomal proteins, including Lamp-1 and cathepsin-B and p21WAF/Cip1 levels decline. 188Re-liposome is effective in the suppression of stemness markers’ expression.	(Chang et al., 2017)
Trehalose	Inhibition; activation	Inhibition of autophagosome and lysosome fusion. Inhibition of cellular import of glucose and fructose through SLC2A (GLUT) transporters; stimulation of autophagy through AMPK and activation of ULK1.	(Lee et al., 2018) (Mardones et al., 2016)
NAD+ (and its precursors)	Activation	Induction of autophagy/mitophagy	(Bonkowski and Sinclair, 2016)
2-deoxyglucose	Activation	ER stress and unfolded protein response; inhibition of glycolysis.	(Stein et al., 2010)

AMPK, 5′ AMP-activated protein kinase; HDAC, histone deacetylase; TCA cycle, tricarboxylic acid cycle; TFEB, master regulator transcription factor EB; SQSTM1/p62, the TFEB regulated adaptor protein; microtubule-associated protein 1A/1B-light chain 3; ER, endoplasmic reticulum, NAD+, nicotinamide adenine nucleotide.

### Clinical Development

Today, published clinical data have demonstrated sufficient evidence for efficacy in various disorders. They also indicate the need for further studies, which is expected to open a new era of autophagy-based therapies. Published clinical trials with or without (only protocol) results are summarized in **Tables 3** and **4**. These studies were searched in the PubMed data base using the key words “phase I/II/III trial OR clinical study” AND “autophagy” (**Tables 3** and **4**) or they were included in the tables following searching in the NCT data base using the key words “autophagy” AND “drug/intervention”. Registered clinical trials listed in the NCT database are summarized in **Table 5**. Most of these clinical studies focus on cancer, but new fields such as infections, neurodegenerative diseases, antiaging, cognitive decline, venous endothelial function, and inflammation reduction in acute coronary syndrome are also under investigation.

**Table 3:** Published clinical trials based on autophagy modulation in neurodegenerative, infectious, and other diseases.

Study Design/Registry Number	Regimen	Indication/Autophagy Biomarker	Indication/Aims–Results/Status	Reference
Phase II R, DB, PC, MC NCT03693781 Eudract n.2017-004459-21	Colchicine 0.01 mg/kg/d; 0.005 mg/kg/d Riluzole: 100 mg/d Treatment: 30 wt Follow-up: 24w	ALS/Quantification of mRNA and protein levels of p62, LC3, TFEB, ATGs, HSPB8, BAG3, BAG1, HSP70 and HSF1 in PBMCs, lymphoblasts and fibroblasts (transcriptome profile)	Efficacy of colchicine on disease progression as measured by ALS Functional Rating Scale - Revised (ALSFRS-R) at baseline and at treatment end. Results are not yet available.	(Mandrioli et al., 2019)
Phase II R, DB, PC, MC Eudract n.2016-002399-28	Sirolimus 1 mg/m^2^/d; 2 mg/m^2^/d	ALS/mTOR downstream pathway (S6RP phosphorylation)	Efficacy of sirolimus in ALS patients on functional rating scale, survival, forced vital capacity, and quality of life. Results are not yet available.	(Mandrioli et al., 2018)
Case report, OL, R N (Test) = 22 N (control) = 20	1,000–1,500 mg metformin plus insulin and anti-TB treatment	Diabetes mellitus and tuberculosis/MAP1LC3B	MET has the potential to enhance the bactericidal effect of antituberculosis (sputum smear reversion after 2 months) *via* autophagy. MAP1LC3B level increased significantly by metformin treatment.	(Novita et al., 2019)
Phase IIa, R, PC, double blind NCT03094546	Spermidine-based nutritional supplementation	Elderly with subjective cognitive decline/LC3 I/II, p62, EP300, proteomics, metabolomics, polyamine levels, metabolomics, proinflammatory biomarkers, and neurotrophin levels	Results are not yet available.	(Wirth et al., 2019)
Phase IIB, OL, RCT CTRI/2018/01/011176	İsoniazid: 150–300 mg/d Rifampicin. 300–600 mg/d Pyrazinamide: 800–1,600 mg/d Ethambutol: 550–1,100 mg/d Test group: plus metformin 1,000 mg/d	Newly diagnosed smear positive pulmonary tuberculosis/immunological and autophagy biomarkers (T cell, monocyte and dendritic cell functions ESAT-6/CFP-10, Culture filtrate Protein, estimation of C reactive protein, tumor necrosis factor-alpha and other cytokines).	Autophagy response will be evaluated as a secondary endpoint. Results are not yet available.	(Padmapriyadarsini et al., 2019)
Phase IIa, R, PC, double blind NCT02755246	Spermidine-rich plant extract supplement	Behavioral mnemonic similarity task/not assessed	Memory performance was moderately enhanced and mnemonic discrimination ability improved in the treatment group versus the placebo group/not assessed	(Wirth et al., 2018)
Phase IV, R, quadruple-blind N = 10 NCT02058173	CQ: 150 mg/d × 8 + 4 w Placebo 8 w	HCV/HCV genotype, IL28 genetic polymorphism	A significant decrease in HCV-RNA after the treatments (week 8) was observed in all patients in the CQ-group. The IL28 polymorphism was not associated with 5 HCV RNA load in response to CQ. Preliminary evidence that CQ is possibly a safe treatment option for HCV nonresponders	(Peymani et al., 2016)

R, randomized; DB, double-blind; OL, open label; PC, placebo-controlled; MC, multicenter; N, number of patients; d, day; w, week; m, month.

**Table 4:** Published clinical trials based on autophagy modulation in cancer.

Study Design/Registry Number	Regimen	Indication/Autophagy Biomarker	Results	Reference
Phase II, R, DB, PC N = 70 NCT02333890	CQ: 500 mg/d Treatment: 14–29 d, 2–6 weeks before surgery	Newly diagnosed breast cancer/not assessed	No significant difference in Ki67 index (a proliferation-associated nuclear antigen).	(Arnaout et al., 2019)
Phase I/II, OL N = 33 NCT01510119	HCQ: 600 mg BID Everolimus:10 mg/d	Clear-cell renal cell carcinoma (previously treated)/not assessed	PR + SD: 67%; PR: 6%; PFS ≥ 6 m: 45%. The primary endpoint (> 40% 6-month PFS rate) was met. HCQ is a tolerable inhibitor of autophagy.	(Haas et al., 2019)
Phase II, Simon’s two-stage design; N = 21 NCT01748500	Pantoprazole: 240 mg/3w Docetaxel: 75 mg/m^2^/3w	mCRPC/not assessed	PR = 31%; mOS = 15.7 m; median PFS = 5.3 m. Tolerable but clinical activity is insufficient.	(Hansen et al., 2019)
Phase Ib/II, Single-arm, OL N = 40 NCT01649947	HCQ: 200 mg BID/1–21 Day 1: Paclitaxel: 200 mg/m^2^ Carboplatin: AUC = 6 Bevacizumab: 15 mg/kg	mNSCLC/not assessed	ORR = 33% (44% in KRAS positive tumors); SD: 53%; PFS: 3.3 m (6.4 m in KRAS positive tumors). Addition of HCQ is safe and tolerable with a modest improvement in clinical responses.	(Malhotra et al., 2019)
Phase I, OL N = 35 NCT01480154	HCQ: 200–600 mg BID MK-2206 135 0r 200 mg/w	Advanced solid tumors/pre-planned autophagy biomarkers are not assessed due to high attrition.	SD: 15%; combination increases HCQ plasma levels. Combination therapy is tolerable but antitumor activity is minimal.	(Mehnert et al., 2019)
Phase 2, R, OL N = 112 NCT01506973	HCQ: 600 mg BID Gemcitabine hydrochloride +nab-paclitaxel (GA) with or without	Pancreatic cancer/not assessed	HCQ: 12 m OS: non-HCQ: 41%; 49%, OS: 11.1 in HCQ; 12.1 in non-HCQ PFS: 5.7 m HCQ; 6.4 m non-HCQ, ORR: 38.2% in HCQ; 21.1% in non-HCQ HCQ did not improve the primary end point of OS but may play a role in the locally advanced setting, where tumor response may permit resection.	(Karasic et al., 2019)
Phase III, OL, R N = 500 NCT03504423 (AVENGER 500 trial)	CPI-613 + modified FOLFIRINOX (devimistat 500 mg*/*m^2^, oxaliplatin 65 mg*/*m^2^, irinotecan dose of 120 mg*/*m^2^; fluorouracil, 400 mg*/*m^2^ (bolus), then 2,400 mg*/*m^2^/2 w FOLFIRINOX (oxaliplatin, 85 mg*/*m^2^; irinotecan, 180 mg*/*m^2^; fluorouracil and leucovorin (same)	Metastatic adenocarcioma of pancreas/mitochondrial SOD2, PDK1-4, PDH, KGDH and CD79a and whole-exome sequencing	Objectives: evaluation of ORR and PFS; tumor response Results are not yet available.	(Philip et al., 2019)
Phase I, OL, cohort N = 14 NCT01687179	HCQ: 100–200 mg BID Sirolimus: 2 mg (trough levels between 5 and 15 ng/ml)	Lymphangioleiomyomatosis/metabolomic profiling of polyamine metabolism 5′-methylthioadenosine and arginine	Upregulation of 5′-methylthioadenosine and arginine in the plasma of patients with LAM	(Tang et al., 2019)
Phase I, OL, cohort N = 14 24 w treatment + 24 w observation NCT01687179	HCQ: 100–200 mg BID Sirolimus: 2 mg (trough levels between 5 and 15 ng/ml)	Lymphangioleiomyomatosis/AXL receptor tyrosine kinase, brain-derived neurotrophic factor (BDNF), cathepsin D, epidermal growth factor receptor (EGFR), human epidermal growth factor receptor 2, insulin, receptor tyrosine protein kinase erbB3, and soluble superoxide dismutase 1	Only BDNF levels changed significantly. A consistent decrease of BDNF levels in comparison to baseline was observed which was not HCQ dosage-dependent.	(Lamattina et al., 2018)
NR, OL N = 30 (healthy) N = 43 (EM)	None	Endometriosis/LC3B-II	The expression of LC3B-II in ectopic endometrium group was significantly lower than that of its eutopic endometrium group. Down-regulated autophagy of ectopic endometrium in secretory phase may be related to the progression of EMs.	(Li et al., 2018a)
Phase I, OL N = 20 NCT01835041	CPI-613 (devimistat): 500 mg/m^2^/d Modified FOLFIRINOX (oxaliplatin at 65 mg/m^2^, leucovorin at 400 mg/m^2^, irinotecan at 140 mg/m^2^, and fluorouracil 400 mg/m^2^ bolus followed by 2,400 mg/m^2^ over 46 h)	Metastatic pancreatic cancer/not assessed	MTD of CPI-613 = 500 mg/m^2^ per day. 18 patients treated at MTD, ORR (CR + PR) = 61%. PFS = 9 m; mOS =19 m. Clinical activity requires Phase II validation.	(Alistar et al., 2017)
Phase III, R 1. N = 219 2. N = 234 3. N = 204 NCT00719797 NCT00433927	1. TRIBE trial (discovery cohort) FOLFIRI (fluorouracil, leucovorin, and irinotecan) plus bevacizumab 2. FIRE-3 trial (validation cohort) FOLFIRI plus bevacizumab 3. FIRE-3 trial (negative control) FOLFIRI plus cetuximab	mCRC/12 SNPs in eight autophagy-related genes were examined in this study (autophagy-related protein 13 [ATG13], ATG3, ATG5, ATG8, beclin 1, FIP200, ULK1 and UVRAG	G allele of the FIP200 rs1129660 SNP showed a significantly lower rate of grades 2–3 hypertension compared with the A/A genotype. Polymorphisms in autophagy-related FIP200 gene may be predictive of hypertension.	(Berger et al., 2017)
OL, case report N = 2 NCT02271516	188Re-liposome (0.42 ± 0.04 mCi/kg)	Recurrent ovarian cancer/Cancer Antigen 125 (CA-125) as a marker of drug-resistance	188Re-liposome reduces CA-125 levels and improves survival.	(Chang et al., 2017)
Phase I, OL, cohort N = 14; 24 w treatment + 24 w observation NCT01687179	HCQ: 100–200 mg BID Sirolimus: 2 mg (trough levels between 5 and 15 ng/ml)	Lymphangioleiomyo-matosis/not assessed	Well tolerated; improvement in lung function at 24 weeks, with a decrease in lung function at the 48-week time point.	(El-Chemaly et al., 2017)
Phase I/II, NR, OL N = 12 NCT01023477	CQ: 250 mg/w and 500 mg/w × 4w	Breast ductal carcinoma *in situ* (DCIS)/LC3B positive puncta	Chloroquine reduces PCNA proliferation index in DCIS lesions and inhibits autophagic flux (LC3B positive puncta)	(Espina et al., 2017)
Phase I/II OL, R N (ricolinostat) = 15 N (combined) = 57	Ricolinostat: Phase I cohorts 1–6: 40, 80, 160, 240, and 360 mg on days 1–5 and 8–12 of each 21-day cycle. Bortezomib: 1.0 mg/m^2^/1.3 mg/m^2^. Dexamethasone	Relapsed or refractory multiple myeloma/not assessed	Ricolinostat of 160 mg daily, the combination with bortezomib and dexamethasone is safe, well tolerated, and active.	(Vogl et al., 2017)
OL, NR N = 65	5-FU based treatment (*In vitro* and *in vivo* clinical data with gossypol (AT-101) are linked to clinical data)	Gastric cancer/APE1 expression	Expression of APE1 is associated with poor survival in gastric cancer patients. AT101, an APE1 inhibitor, may promote chemotherapeutic sensitivity.	(Wei et al., 2016)
Phase I, OL, NR N = 20 NCT01023737	HCQ: 400 mg/day Vorinostat: 600 mg/day	mCRC/CTSD and LC3-II in on study-biopsies	SD > 16w: 26%; mPFS 2.8 m; mOS 6.7 m. Improved antitumor immunity (decreased exhausted and regulatory T cells and increased effector phenotype T cells) and reduced tumor autophagy	(Patel et al., 2016)
Phase Ib/OL, NR N = 38 NCT01583283	Ricolinostat: 40–320 mg/d Lenalidomide: 15–25 mg/d Dexamethasone: 40 mg/w	Relapsed or refractory multiple myeloma/HDAC6	DLT: ricolinostat ricolinostat 160 mg BID. The pharmacokinetics of ricolinostat and lenalidomide were not affected by coadministration. ORR:55%	(Yee et al., 2016)
Phase I, OL, NR N = 25	HCQ: 400 mg/d Sirolimus: 2 mg/d Metronomic chemotherapy	Stage IV refractory metastatic solid tumors/not assessed	ORR=40%; SD:84% Tumor markers dropped >50%. Progression from PD to PR:2; SD to PR:8 Autophagy is a promising target and warrants further Phase II studies	(Chi et al., 2015a)
Phase II, OL, single arm N = 10 NCT01842594	Sirolimus: 1 mg HCQ: 200 mg 2×1/d × 2 w	Sarcoma/uptake of [^18^F]-fluorodeoxyglucose positron emission tomography (FDG PET)	An inhibition of glycolysis within the tumors without tumor growth was noted. PR: 6/10; SD:3/10, PD:1/10.	(Chi et al., 2015b)
Phase II, OL N = 19 (evaluable) NCT01206530	12 cycles:HCQ: 600 mg BID+ FOLFOX (5-FU (400 mg/m^2^ bolus, then 2,400 mg/m^2^ over 46h) + leuco-vorin 200 mg/m^2^, oxaliplatin 85 mg/m^2^)/bevacizumab 5 mg/kg, all iv/2 w; after 12 cycles, no oxaliplatin.	Previously untreated mRCR/autophagy biomarkers in PBMC	Autophagy is inhibited in PBMCs. ORR: 52% (CR: 5%, PR: 47%) FOLFOX/bevacizumab + HCQ is an active regimen in mCRC.	(Loaiza-Bonilla et al., 2015)
Phase I/II, OL, NR N = 35 NCT01128296	HCQ: 1200 mg/d for 31 d Gemcitabine: 1,500 mg/m^2^ on days 3 and 17	Pancreatic adenocarcinoma/LC3-II in PBMC	No dose-limiting toxicities and no Grade 4/5 events related to treatment. 61% had a decrease in CA19-9. Patients with a >51% increase of LC3-II in PBMC had improvement in PFS (15.03 *vs.* 6.9 months, p < 0.05) and OS (34.8 *vs.* 10.8 m, p < 0.05). Preoperative autophagy inhibition with HCQ plus gemcitabine is safe and well tolerated. Surrogate biomarker responses (CA 19-9) and surgical oncologic outcomes were encouraging.	(Boone et al., 2015)
Case series, OL, NR N = 5	CQ: 250 mg/d Reirradiation	Recurrent glioblastoma	No CQ related toxicity. 2 PR, 1 SD, 1 PD. Encouraging responses were obtained.	(Bilger et al., 2014)
Phase I, OL N = 24	Pantoprazole: 80, 160, 240, and 360 mg *iv* prior to doxorubucin (60 mg/m2)	Solid tumors/not assessed	Pantoprazole 240 mg with doxorubicin 60 mg/m^2^ every 3 weeks: toxicity was predictable and manageable.	(Brana et al., 2014)
Phase I, OL, cohort N = 24 NCT01023737	HCQ: 400–800 mg/d (d2–d21) Vorinostat: 400 mg/d (d1–21)	Advanced solid tumors/AV, lysosomal protease CTSD, CDKN1A	HCQ and VOR stimulate the expression of CTSD and CDKN1A and the accumulation of autophagic vacuoles in PBMC. HCQ addition had no significant impact on the pharmaco-kinetic profile of VOR. 46% had PR or SD for ≥2 cycles. Based on the safety and preliminary efficacy of this combination, additional clinical studies are currently being planned	(Mahalingam et al., 2014)
Phase Ib/II, OL N = 27	HCQ: 1200 mg/d Temsirolimus: 25 mg/w	Advanced solid tumors and melanoma/AV accumulation in PBMC.	Significant AV accumulation with TEM + HCQ compared with baseline only with 1200 mg cohort. SD = 74%; further studies are warranted. TEM and HCQ: safe and tolerable, modulate autophagy in patients, and have significant antitumor activity.	(Rangwala et al., 2014a)
Phase Ib/II, OL N = 40	HCQ:1200 mg/d Temozolomide: 150 mg/m^2^ daily for 7/14 d	Advanced solid tumors and melanoma/autophagic vacuoles peripheral blood mononuclear cells	PR = 14%; SD: 27% in 22 evaluable patients with advanced melanoma. Prolonged stable disease and responses suggest antitumor activity in melanoma patients.	(Rangwala et al., 2014b)
Phase I/II OL N (Phase I) = 16 N (Phase II) = 76 NCT00486603	HCQ: 200–800 mg/d Temozolomide: initially before RT-75 mg/m(2); maintenance-150 mg/m(2)/d; 5 d/m for 6 m. RT: 60 Gy in 30 fractions	Newly diagnosed gliablastoma multiforme/AV and LC3-II	MTD of HCQ: 600 mg/d. 800 mg/d: Grades 3–4 neutropenia and thrombocytopenia; OS 12 m = 70%; OS 18 m = 36%; OS 24 m = 25%. HCQ-induced dose dependent increases in AV and LC3-II in PBMC. Autophagy was not consistently achieved. No significant improvement in overall survival.	(Rosenfeld et al., 2014)
Phase Ib/II, OL N = 25	HCQ: 1,200 mg/d Bortezomib: 1.3 mg/m^2^	Relapsed/refractory myeloma/AV accumulation and LC3-II in PBMC	Therapy-induced AV accumulation in bone marrow plasma cells. PR = 14%, MR = 14%, SD = 45%; further studies are warranted.	(Vogl et al., 2014)
Phase II OL N = 20 NCT01273805	HCQ: 800 and 1,200 mg/d Previously treated with other regimens	Metastatic pancreatic adenocarcinoma (previously treated)/LC3-II in lymphocytes	Analysis of LC3-II showed inconsistent autophagy inhibition. SD: 10%; PFS: 46.5 d; OS: 69 d. Negligible therapeutic efficacy.	(Wolpin et al., 2014)
Phase II R, PC, DB N = 73 NCT01894633	CQ: 150 mg/d × 4 w WBI (30 Gy in 10 fractions/d over 2 weeks)	Brain metastases from solid tumors/not assessed	ORR = CLQ-54%; PL-55%. PFS = CLQ-84%; PL-55% CLQ + WBI improved the control of brain metastasis with no increase in toxicity. CLQ did not improve the RR or OS.	(Rojas-Puentes et al., 2013)
Pilot, single cohort N = 20	CQ: 250 mg/day × 5 w; started 1 w before WBRT	Newly diagnosed brain metastases from solid tumors	CR = 2; PR = 12; SD = 1 No treatment-related grade ≥ 3 toxicities or treatment interruption due to toxicity. Median/mean OS = 5.7 and 8.9 m	(Eldredge et al., 2013)
Phase Ib/II, OL N = 12	2-deoxyglucose (2-DG) 45 mg/kg	mCRPC/p62 as marker of 2-DG resistance	P62 decreased in 83% and fluorodeoxyglucose uptake decreased in 63% of patients. 2-DG alone or in combination can be used to target tumor metabolism.	(Stein et al., 2010)
Phase II, OL N = 30	Sorafenib: 400 mg × 2/d	Relapsed or refractory lymphoma/ LC3‐II in PBLs	ORR: 13%; OS: 16 m. LC3-II levels at baseline were signiﬁcantly higher in responsive patients than in nonresponsive patients. PBLs: responsive patients: reduction in LC3-II levels; nonresponsive patients: no change.	(Guidetti et al., 2012)
Phase I, OL, R N(HCQ) = 27 N(HCQ + Erlotinib) = 19 NCT01026844	HCQ: 400–1000 mg/d (escalated) Erlotinib: 150 mg/d	Advanced NSCLC/not assessed	HCQ with or without erlotinib was safe and well-tolerated. The recommended phase 2 dose: HCQ 1000mg + erlotinib 150mg.	(Goldberg et al., 2012)
Phase II, R, DB, PC N = 30 NCT00224978	CQ: 150 mg/d Conventional chemotherapy + radiotherapy	Glioblastoma multiforme	OS = 24m for CQ; 11m for PL Small sample size suggests larger, more definitive studies.	(Sotelo et al., 2006)

R, randomized; DB, double-blind; OL, open label; PC, placebo-controlled; MC, multicenter; N, number of patients; d, day; w, week; m, month; RT, Radiotherapy; PL, placebo; HCQ, hydroxychloroquine; CQ, chloroquine; OS, overall survival; ORR, objective response rate; CR, complete response; PR, partial response; MR, minor response; SD, stable disease; PFS, progression-free survival.

mCRPC, metastatic castration-resistant prostate cancer; mNSCLC, metastatic nonsmall cell lung cancer; mCRC, metastatic colorectal cancer; PBMC, peripheral blood mononuclear cells; AV, autophagic vacuole; UVRAG, UV radiation resistance-associated gene protein; FIP200, focal adhesion kinase family interacting protein of 200 kDa; ULK1, unc-51-like kinase 1; CDKN1A, cyclin-dependent kinase inhibitor 1A.

**Table 5:** Registered clinical trials based on autophagy modulation in various diseases.

NCT number	Study Design	Title	Regimen	Study Start/Study End	Result
NCT04160455	Observational model: cohort	Study of autophagy and the effects of GALIG gene products in HIV-1 infected patients who are under antiretroviral therapy since primary-infection, chronic phase, or never treated.	HIV-1 infected patients under antiretroviral therapy since primary-infection, chronic phase, or never treated	November 7, 2019/November 7, 2029	Recruiting
NCT04138134	Observational model: cohort	Autophagy and venous endothelial function	Spermidine	December 1, 2019/June 1, 2020	Not yet recruiting
NCT03979651	NR, OL	MEK and autophagy inhibition in metastatic/locally advanced, unresectable neuroblastoma RAS (NRAS) melanoma	Trametinib plus HCQ	September 30, 2019/March 31, 2022	Ongoing
NCT00786682	Phase II, NR, OL	Docetaxel and hydroxychloroquine in treating patients with metastatic prostate cancer	Docetaxel and HCQ	December 2008–October 2012	The study was stopped due to lack of improved efficacy compared to historical controls.
NCT00765765	Phase I/II, NR, OL	Ixabepilone and hydroxychloro-quine in treating patients with metastatic breast cancer	Ixabepilone and HCQ	February 2009–December 2012	The study was closed early due to slow accrual
NCT00969306	Phase I, NR, OL	Chloroquine as an antiautophagy drug in stage IV small cell lung cancer (SCLC) patients	Chloroquine	September 2013–June 2016	Terminated but no results posted
NCT03037437	Phase II, NR, OL	Sorafenib induced autophagy using hydroxychloroquine in hepatocellular cancer	Sorafenib + HCQ	February 16, 2017–December 2020	Ongoing
NCT01480154	Phase I, NR, OL	Akt inhibitor MK2206 and hydroxychloroquine in treating patients with advanced solid tumors, melanoma, prostate or kidney cancer	MK2206 + HCQ	November 23, 2011–December 2019	(Mehnert et al., 2019)
NCT01510119	Phase I/II, NR, OL	Autophagy inhibition to augment mTOR Inhibition: A Phase I/II Trial of RAD001 and hydroxychloroquine in patients with previously treated renal cell carcinoma	RAD001 (everolimus) + HCQ	September 2011–January 2017	(Haas et al., 2019)
NCT01023737	Phase I, NR, OL	Hydroxychloroquine + Vorinostat in advanced solid tumors	HCQ + Vorinostat	November 2009–September 2020	(Patel et al., 2016)
NCT03598595	Phase I/II, NR, OL	Gemcitabine, docetaxel, and hydroxychloroquine in treating participants with recurrent or refractory osteosarcoma	Gemcitabine, Docetaxel, and HCQ	January 28, 2019–March 2, 2020	Ongoing
NCT02631252	Phase I, NR, OL	Phase I Study of mitoxantrone and etoposide combined with hydroxychloroquine, for relapsed acute myelogenous leukemia	Mitoxantrone and Etoposide and HCQ	August 18, 2016–October 2, 2017	Terminated due to inability to accrue.
NCT03774472	Phase I, NR, OL	Hydroxychloroquine, palbociclib, and letrozole before surgery in treating participants with estrogen receptor positive, HER2 negative breast cancer	HCQ, Palbociclib, and Letrozole	August 20, 2018–December 31, 2020	Ongoing
NCT02339168	Phase I, NR, OL	Enzalutamide and metformin-hydrochloride in treating patients with hormone-resistant prostate cancer	Enzalutamide and Metformin-Hydrochloride	June 2016–July 2020	Ongoing
NCT01594242	Phase I, NR, OL	Autophagy induction after bortezomib for myeloma	Bortezomib	July 10, 2012–March 2, 2015	Completed but no results are available
NCT03700424	Phase II, R, quadruple-blind	Inflammation reduction by Trehalose administration (in acute coronary syndrome)	Trehalose vs normal saline infusion weekly (15 g/week) for a period of 12 weeks	July 7, 2019–June 2020	Ongoing
NCT01266057	Phase I, NR, OL	Sirolimus or vorinostat and hydroxychloroquine in advanced cancer	Sirolimus or Vorinostat and HCQ	April 28, 2011–February 2021	Ongoing
NCT02378532	Phase I, NR, OL	The addition of chloroquine to chemoradiation for glioblastoma	Chloroquine + temozolomide + chemoradiation	August 2016–June 2019	Ongoing (still recruiting)
NCT01006369	Phase II, NR, OL	Hydroxychloroquine, capecitabine, oxaliplatin, and bevacizumab in treating patients with metastatic colorectal cancer	HCQ, Capecitabine, Oxaliplatin, and Bevacizumab	May 2009–April 27, 2016	Completed but results are not yet available
NCT03309007	Phase III, R, quadruple-blind	A double-blind, placebo-controlled trial of anti-aging, pro-autophagy effects of metformin in adults with prediabetes	Metformin Placebo	September 1, 2017–July 31, 2021	Active, not recruiting
NCT02042989	Phase I, NR, OL	MLN9708 and Vorinostat in patients with advanced p53 mutant malignancies	MLN9708 and Vorinostat	June 27, 2014–June 2022	Active, not recruiting
NCT00728845	Phase I/II, NR, OL	Hydroxychloroquine, carboplatin, paclitaxel, and bevacizumab in recurrent advanced nonsmall cell lung cancer	HCQ, Carboplatin, Paclitaxel, and Bevacizumab	June 16, 2008–December 21, 2010	Terminated due to slow accrual
NCT03754179	Phase I/II, R, OL	Dabrafenib/trametinib/hydroxychloroquine for advanced pretreated BRAF V600 mutant melanoma	Dabrafenib/Trametinib/HCQ	January 23, 2018–December 2020	Recruiting
NCT02316340	Phase II, R, OL	Vorinostat Plus hydroxychloroquine *vs* regorafenib in colorectal cancer	Vorinostat + HCQ *vs* Regorafenib	February 2015–September 2020	Active, not recruiting
NCT02432417	Phase II, R, OL	The addition of chloroquine to chemoradiation for glioblastoma,	Chloroquine + Chemoradiation	January 2020–January 2024	Not yet recruiting
NCT02257424	Phase I/II, NR, OL	Dabrafenib, trametinib, and hydroxychloroquine in patients with advanced BRAF mutant melanoma	Dabrafenib, Trametinib and HCQ	October 2014–October 2026	Active, recruiting
NCT02421575	Phase I, NR, OL	Hydroxychloroquine in blocking autophagy in patients with prostate cancer undergoing surgery or active surveillance	HCQ	July 2012–February 26, 2016	Terminated due to slow accrual
NCT01206530	Phase I/II	FOLFOX/Bevacizumab/Hydroxychloroquine (HCQ) in Colorectal Cancer	HCQ, Oxaliplatin, Leuco-vorin, 5-fluorouracil, Bevacizumab	September 2010 –September 2017	(Loaiza-Bonilla et al., 2015)
NCT00962845	Phase I, NR, OL	Hydroxychloroquine in patients with stage III or stage IV melanoma that can be removed by surgery	HCQ	September 2010–May 2013	Early Phase I is completed. Last update September, 2018. https://ichgcp.net/es/clinical-trials-registry/NCT00962845
NCT01144169	Phase I, NR, O	Study of hydroxychloroquine before surgery in patients with primary renal Cell carcinoma	HCQ	October 2010–September 2016	Terminated due to barriers to accrual such as delay until surgery and additional preoperative visits
NCT03094546	Phase III, R, triple-blind	Polyamine-enriched diet in elderly individuals with subjective cognitive decline	Polyamine-rich diet	June 2019 – June 2020	(Wirth et al., 2019)
NCT01583283	A Phase I/II, Open Label, Multi-center Study	Study of ACY-1215 in combination with lenalidomide, and dexamethasone in multiple myeloma	Ricolinostat (*ACY*-*1215*) Lenalidomide Dexamethasone	July 12, 2012–December 31, 2019	(Yee et al., 2016)
NCT01997840	Phase 1B/2 Multi-Center, Open Label, Dose-escalation Study	ACY-1215 (ricolinostat) in combination with pomalidomide and low-dose dex in relapsed-and-refractory multiple myeloma	ACY-1215 (ricolinostat) in combination with pomalidomide and dexamethasone	March 1, 2014–January 27, 2021	Active, not recruiting
NCT02189343	Phase 1b Multi-Center, Open Label, Dose-Escalation Study	Phase 1b Study evaluating ACY-1215 (ricolinostat) in combination with pomalidomide and dexamethasone in relapsed or relapsed-and-refractory multiple myeloma	ACY-1215 (Ricolinostat) Pomalidomide Dexamethasone	September 15, 2014–January 30, 2019	Active, not recruiting

R, randomized; DB, double-blind; OL, open label; PC, placebo-controlled; MC, multicenter; N, number of patients; d, day; w, week, m, month; RT, Radiotherapy; PL, placebo; HCQ, hydroxychloroquine; CQ, chloroquine; OS, overall survival; ORR, objective response rate; CR, complete response; PR, partial response; MR, minor response; SD, stable disease; PFS, progression-free survival.

As seen in **Tables 3** and **4**, the role of autophagy in the prognosis of various diseases received increasing interest, especially in the last decade. Various biomarkers were utilized in these clinical trials to measure autophagy response. In the early studies, autophagic vacuoles were evaluated by transmission electron microscopy, but this method was unreliable (Levy et al., 2017). Selected biomarkers of autophagy include microtubule associated protein light chain 3 (LC3), p62, ATGs in peripheral blood mononuclear cells (PBMCs), lymphoblasts, and fibroblasts of patients. Proteomics, metabolomics, lysosomal protease cathepsin D (CTSD), cyclin-dependent kinase inhibitor 1 (CDKN1A, p21/cip1/waf1), and S6RP phosphorylation are among the potential biomarkers considered.

### Cancer

Cancer is the extensively studied field in autophagy (**Table 4**). CQ and HCQ are mostly included in treatment regimens to increase the sensitivity of chemotherapeutics. CQ treatment at a relatively high daily dose of 500 mg resulted in no significant difference in the classic cellular proliferation marker, Ki67 index, in newly diagnosed breast cancer patients in a Phase II, double-blind, randomized trial (Arnaout et al., 2019). CQ at a daily dose of 150 mg was able to reduce the proliferating cell nuclear antigen (PCNA) index in breast ductal carcinoma (Espina et al., 2017). CQ at 150 mg/day together with conventional chemotherapy plus radiotherapy improved overall survival when compared to placebo in glioblastoma multiforme (GBM), but the small sample size of 30 patients was not sufficient for reaching a conclusion (Sotelo et al., 2006). A higher dose of CQ (250 mg/day) added to radiotherapy gave encouraging results in a pilot study of five patients with GBM (Bilger et al., 2014). A small size study, which enrolled newly diagnosed brain metastasis patients, showed that CQ at 250 mg/day, starting 1 week before radiotherapy and continuing for 5 weeks, was well tolerated with a PR rate of 60% (Eldredge et al., 2013). In comparison, CQ at a daily dose of 150 mg for four weeks plus radiotherapy, when compared to placebo, also improved brain metastasis control and prolonged progression free survival (PFS),but achieved no benefit in terms of response rate or overall survival (Rojas-Puentes et al., 2013).

Combination of HCQ with mTOR inhibitors, which enhance cytoprotective autophagy, was tested in Phase I/IIa trials. HCQ in combination with everolimus was tolerable and the primary endpoint of a 6-month progression free survival (PFS) rate over 40% was met in clear cell renal carcinoma (Haas et al., 2019). HCQ in combination with sirolimus significantly increased the “forced expiratory volume” but not the “forced vital capacity” in lymphangioleiomyomatosis (El-Chemaly et al., 2017). Metabolomic profiling of polyamine metabolism (upregulation of 5′-methylthioadenosine) and brain derived neurotrophic factor were suggested as candidate markers associated with autophagy in lymphangioleiomyomatosis (Lamattina et al., 2018; Tang et al., 2019). HCQ combined with temsirolimus showed significant antitumor activity in advanced solid tumors and melanoma (Rangwala et al., 2014a). HCQ and sirolimus decreased glycolysis mainly in cancer associated fibroblasts, hence attenuated their metabolic-parasite relationship with sarcoma cells; this double autophagy modulation resulted in an overall response rate (ORR) of 90% (Chi et al., 2015b). Addition of sirolimus and HCQ to the ongoing metronomic chemotherapy, to which patients were previously unresponsive, resulted in high salvage rates in stage IV refractory metastatic tumors (Chi et al., 2015a).

HCQ alone or in combination provided minimal to modest benefit with the EGFR inhibitor erlotinib in metastatic nonsmall cell lung cancer (Goldberg et al., 2012; Malhotra et al., 2019); with FOLFOX/bevacizumab in metastatic colorectal cancer (Loaiza-Bonilla et al., 2015); with temozolomide in advanced solid tumors and melanoma (Rangwala et al., 2014b); with vorinostat (Mahalingam et al., 2014; Patel et al., 2016) and MK-2206 (Mehnert et al., 2019) in advanced solid tumors; as a single agent (Wolpin et al., 2014), with gemcitabine (Boone et al., 2015) and with gemcitabine plus nab-paclitaxel (Karasic et al., 2019) in advanced pancreatic cancer; with temozolomide plus radiotherapy in newly diagnosed glioblastoma multiforme (Rosenfeld et al., 2014) and with bortezomib in refractory myeloma (Vogl et al., 2014).

Ricolinostat, a selective HDAC6 inhibitor, was well tolerated and moderately effective in combination with bortezomib (Vogl et al., 2017) and the immunomodulator lenalidomide plus dexamethasone (Yee et al., 2016) in refractory multiple myeloma.

The proton pump inhibitor, pantoprazole, was tolerable when combined with doxorubicin in solid tumors (Brana et al., 2014) but showed insufficient clinical activity when combined with docetaxel in metastatic castration resistant prostate cancer (mCRPC) (Hansen et al., 2019).

Two novel drugs seem to be promising as autophagy inhibitors. Devimistat (CPI-613) inhibits two key enzymes of the tricarboxcylic acid cycle, *i.e.* pyruvate dehydrogenase and ketoglutarate dehydrogenase, and thus impairs pancreatic cell mitochondrial metabolism. Devimistat, in combination with the FOLFIRINOX regimen, exhibited an ORR of 61% (Alistar et al., 2017) in a Phase I trial and is also under evaluation in a large scale Phase III trial (AVENGAR 500) in metastatic pancreas adenocacinoma (Philip et al., 2019). ^188^Re-liposome treatment was successful in inducing mitochondrial autophagy and improved survival in two cases with recurrent ovarian cancer (Chang et al., 2017).

Natural products were also investigated as autophagy modulators. Spermidine, a natural polyamine, triggers autophagy by inducing the acetylation or deacetylation of autophagy-related genes (Yang et al., 2017). Spermidine-based nutritional supplementation, leading to the acetylation or deacetylation of autophagy related genes, is considered as an autophagy-based strategy against memory under-performance, mnemonic discrimination ability and cognitive decline in the elderly (Wirth et al., 2018; Wirth et al., 2019). Gossypol (AT-101) is a polyphenolic compound extracted from cotton. In addition to its contraceptive and anti-infective effects, its roles as an anticancer agent by targeting apoptotic and autophagic pathways have also been studied in the last decade. It is a pan-Bcl-2 inhibitor and induces autophagy by liberating Beclin-1 from Bcl2 (Perez-Hernandez et al., 2019). Most of the clinical studies in PubMed focused on its apoptotic effect; only Wei et al. reported that the expression of apurinic/apyrimidinic endonuclease (APE1), which regulates the DNA base excision repair process, reduces survival in gastric cancer patients and the APE1 inhibitor AT-101 may be a potential sensitizer for 5-fluorouracil (Wei et al., 2016). Curcumin, the major constituent of Curcuma longa (turmeric), and quercetin, a plant flavonol, are two other autophagy inducers, which inhibit the Akt/mTOR pathway. No phase trial on autophagy is yet registered with the above-mentioned natural compounds in the NCT database, except for spermidine (Kondapuram et al., 2019; Perez-Hernandez et al., 2019).

Autophagy related polymorphisms might be predictive of anticancer treatment associated adverse effects. Berger et al. reported that FIP200 gene polymorphisms might predict bevacizumab-induced hypertension (Berger et al., 2017).

In summary, the application of autophagy inducers/inhibitors in cancer treatment is a quickly growing area of research, which has the aim of balancing the potentially good and bad effects. The success in keeping this balance will determine the future development and use of such compounds.

### Neurodegenerative Disorders

In amyotrophic lateral sclerosis (ALS), degradation of mutant protein aggregates and damaged organelles is disrupted. Two autophagy modulators, colchicine, which upregulates autophagy-related proteins in motor neurons in combination with riluzole (Mandrioli et al., 2019), and the mTOR inhibitor rapamycin (sirolimus) (Mandrioli et al., 2018) are being evaluated for their therapeutic efficacy against ALS in two separate double-blind, randomized, placebo-controlled phase-II trials.

### Infectious Diseases

The mycobacterium tuberculosis virulence is associated with perturbations in the autophagy process and AMPK signaling. Novita et al. showed that metformin enhances the bactericidal effect of anti-tuberculosis treatment *via* autophagy in patients with tuberculosis and diabetes (Novita et al., 2019). Another study was designed to assess the effect of metformin by means of a detailed autophagy biomarker panel in tuberculosis (Padmapriyadarsini et al., 2019). Chloroquine significantly decreased HCV-RNA in Hepatitis C in a quadruple-blind, randomized Phase IV study (Peymani et al., 2016).

## Alkylphosphocholines

**Figure 3:**
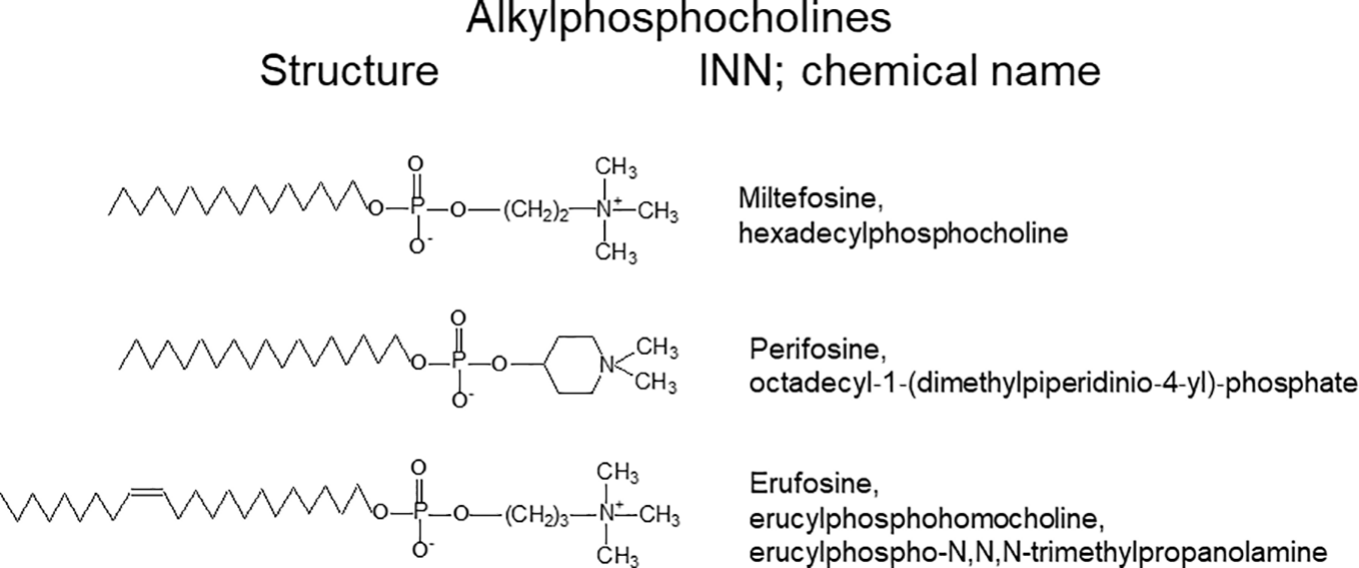
Structures and names of alkylphosphocholines.

Synthetic APCs include miltefosine, perifosine, and erufosine, which correspond to the first, second, and third generations of APCs (see **Figure 3**). They are derived from alkylphospholipids (ALPs) or ‘synthetic antitumor lipids’, which include edelfosine and ilmofosine. The two related groups of antitumor agents target cell membranes rather than DNA (Van Blitterswijk and Verheij, 2013). ALPs are metabolically stable, nonmutagenic and versatile drugs derived from lysophosphatidylcholine (Kaleagasioglu et al., 2019; Zaremberg et al., 2019). All agents possess long hydrocarbon chains that slow their catabolism (Van Blitterswijk and Verheij, 2008).

Cancer researchers became interested in APCs during the 80s of the last century for their selective and high anticancer efficacy in autochthonous, methylnitrosourea-induced rat mammary carcinoma (Berger et al., 1987; Muschiol et al., 1987), which was later confirmed in 7,12-dimethylbenzanthracene-induced rat mammary carcinoma (Hilgard et al., 1988). This unusual spectrum of activity pointed to an influence of APCs on the ras signaling chain, as both chemically induced models exhibit point mutations in the ras gene as driving force for tumor development. In subsequent years, this assumption was confirmed (Berger et al., 1992; Berger et al., 2003; Dineva et al., 2012). Apart from breast cancer models, tumor cell lines from other cancer types were sensitive to ALPs, as well (Erdlenbruch et al., 1998; Jendrossek et al., 1999; Konstantinov and Berger, 1999; Georgieva et al., 2002).

The first generation of APCs, the prototype of which is miltefosine, possesses a typical phosphocholine polar moiety and a saturated alkyl chain of varying length (Berger et al., 1987; Berger et al., 1993). The second generation includes perifosine, which has a cyclic polar head and saturated alkyl chain (Hilgard et al., 1997). Finally, the third generation possesses a classic or modified phosphocholine polar moiety and an unsaturated alkyl chain. The best-known representative of this group is erufosine (Kaleagasioglu et al., 2019).

An ether linkage to its glycerol backbone characterizes the prototype of ALPs, edelfosine (Kaleagasioglu et al., 2019; Zaremberg et al., 2019). The first APC generation is represented by miltefosine (hexadecylphosphocholine, HPC), which lacks the glycerol backbone and thus shows a less complex metabolism (Kaleagasioglu et al., 2019; Zaremberg et al., 2019). In order to reduce the acetylcholine-like side effects observed for derivatives with a phosphocholine headgroup, as miltefosine, perifosine contained a piperidine moiety instead of phosphocholine and thus showed less side effects in clinical applications. Erufosine represents the most novel generation of APCs, which are characterized by a chain length of 22 carbons and the presence of a single double bond (Fiegl et al., 2008; Konigs et al., 2010; Kaleagasioglu and Berger, 2014; Kaleagasioglu et al., 2019; Zaremberg et al., 2019). At clinical concentrations, APCs disturb cell membranes and hamper their phospholipid turnover as well as lipid-based signal and transduction pathways. They may interfere with signaling by hindering the contact of proteins with other cell membrane proteins, or with distinct membrane lipids or lipid microdomains. At high concentrations, they may cause cell lysis owing to their detergent properties (Van Blitterswijk and Verheij, 2013). APCs hinder the normal lipid metabolism and lipid dependent signal transduction by their accumulation in cell membranes. Therefore, they can induce apoptosis in rapidly growing cells, such as tumor cells. They can lead to selective reduction of tumor metastases, impairment of angiogenesis, differentiation of tumor cells, impediment of cytokinesis, cell cycle halt, immune stimulation and intensification of immune reactions against tumors (Van Blitterswijk and Verheij, 2008; Kostadinova et al., 2015; Kaleagasioglu et al., 2019; Zaremberg et al., 2019). Among their mechanism of actions are interference with phospholipid metabolism, the inhibition of survival pathways and alteration of signal transduction (*e.g.* SAPK/JNK AKT-mTOR Ras/Raf, PKC), activation of pro-apoptotic signaling through ALP-triggered stress, and ALP-stimulated Fas/CD95 assembly in lipid rafts (Van Blitterswijk and Verheij, 2013; Kostadinova et al., 2015). This group of drugs interferes with cell division without inhibiting nuclear division, causing increased percentages of cells in the G2/M phase of the cell cycle, with multinucleate cell formation, and ensuing apoptotic cell death (Kostadinova et al., 2015).

### Modulation of Phosphatidylinositol 3-Kinase/Akt/mTOR Signaling by APCs

The PI3K/Akt/mTOR pathway is an important regulator of cell survival, which is activated by receptor tyrosine kinases (RTKs). Following stimulation by growth factors, dimerization of the RTKs triggers an autophosphorylation process and activates PI3K. PIP2 (phosphatidylinositol-3,4-bisphosphate) is then converted to PIP3 (phosphatidylinositol-3,4,5-triphosphate) by PI3K (Zhang, 2004). PI3K signaling is inhibited through the de-phosphorylation of PIP3 by PTEN (tumor suppressor phosphatase and Tensin homolog deleted on chromosome 10) (Liu et al., 2009). PIP3 binds to the pleckstrin homology (PH) domain of the serine/threonine kinase Akt, leading to its allosterical activation. At the next step, phosphorylated Akt (p-Akt) activates mTOR, the key inhibitor of autophagy. Therefore, APCs, by inhibiting Akt, induce autophagy *via* Akt/mTOR signaling pathway (**Figure 2**) (Kaleagasıoglu et al., 2019). Additionally, perifosine inhibits mTOR signaling by promoting degradation of mTOR, raptor, and rictor (Sun, 2010).

Miltefosine inhibits phosphorylation of Akt at its Ser^473^ residue in A431 epidermoid carcinoma cells, which were stimulated by insulin (Ruiter et al., 2003). The related PI3K/Akt signaling has also been implicated in various viral infections. Miltefosine inhibits Chikungunya virus replication by inhibiting Akt phosphorylation in human primary dermal fibroblasts (Sharma et al., 2018). Primary effusion lymphoma (PEL) displays activated PI3K/Akt/mTOR signaling. Both perifosine and miltefosine inhibit proliferation of PEL cell lines and retarded PEL tumor progression *in vivo* (Bhatt et al., 2010).

Perifosine prevents membrane recruitment of Akt and displaces PIP2 and PIP3 from this enzyme. As a result, Akt can no longer proceed through the required conformational change, for its dual phosphorylation, hence activation (Van Blitterswijk and Verheij, 2013). Perifosine blocks phosphorylation of constitutively active Akt within 3 h and also S6 kinase within 6 h in PTEN-mutant human glioma cell lines T98G and U373MG (Momota et al., 2005). Perifosine decreases phosphorylated Akt levels in a dose- and time-dependent manner in the aggressive thyroid cancer cell line FTC133 that has genetic alterations in the PI3K/Akt pathway (Liu et al., 2009). Inhibition of aberrant PI3K/Akt signaling by perifosine in various cancer types including thyroid cancer (Liu et al., 2009), renal cell carcinoma (Porta and Figlin, 2009), neuroblastoma (Sun and Modak, 2012), neuroendocrine tumors (Zitzmann et al., 2012), mantle cell lymphoma (Reis-Sobreiro et al., 2013), and multiple myeloma (Cirstea et al., 2010) seems to be a promising therapeutic strategy. Perifosine also plays a protective role against kainic acid-induced epileptogenesis, which involves abnormal activity of the mTOR pathway. Perifosine pretreatment significantly decreases p-Akt/Akt and p-S6/S6 ratios which results in marked decrease of spontaneous seizure frequency and neuronal death (Zhu et al., 2018)

Erufosine can be infused systemically and shows penetration through the blood brain barrier (Erdlenbruch et al., 1999; Martelli et al., 2010). Erufosine inhibits the aberrant PI3K/Akt/mTOR signaling pathway in various cancer types including leukemia, oral squamous cell carcinoma, glioma and prostate cancer (Erdlenbruch et al., 1998; Rudner et al., 2010; Kapoor et al., 2012; Awde et al., 2013; Kapoor et al., 2014; Ansari et al., 2017; Avsar Abdik et al., 2019). Remarkably, erufosine was less toxic to normal bone marrow than to cancer cells (Bagley et al., 2011; Yosifov et al., 2011). Phosphorylation of PKB/Akt is linked to several proliferation pathways, which are inhibited, correspondingly [for overview see (Kaleagasioglu et al., 2019)]. Erufosine prevents the Akt–mTOR pathway reactivation and development of drug resistance because it targets this pathway at multiple levels. Firstly, it induces dephosphorylation of PKB/Akt at Ser^473^, thus inhibiting the mTORC2 complex. Furthermore, it reduces the phosphorylation of PKB/Akt at its Thr^308^ residue, the upstream kinase activator of the mTORC1 complex. In addition, erufosine causes a dose-dependent decrease in p-PTEN levels, with total PTEN levels remaining unaffected, dephosphorylation of p-mTOR at Ser^2448^, with total mTOR expression levels remaining unaffected. Erufosine also dephosphorylates other constituents of the mTORC1 complex, such as p-PRAS40 and p-Raptor in a concentration-dependent manner, as well as the downstream substrates of mTORC1, *i.e.* p70S6K and p-4EBP1. Knockdown of mTOR by siRNA enhances the cytotoxic potential of erufosine (Kapoor et al., 2014). The effects of erufosine on mTOR explain the dose-dependent induction of autophagy observed with this drug. In addition to these effects, erufosine induces cell cycle arrest in combination with its activity on the Rb gene (Zaharieva et al., 2014; Ansari et al., 2018b).

### Autophagy and Alkylphosphocholines

#### Miltefosine

Miltefosine, the first generation APC, is the only oral drug used for the treatment of leishmaniasis. In trypanosomatids, the shift in balance between posttranslational modifications, polyglutamylation, and deglutamylation may determine cell death and autophagic survival, respectively. Overexpression of polyglutamylases increases miltefosine-induced cell-death, whereas overexpression of deglutamylases, CCP5A and CCP5B, increases miltefosine-induced cell survival process of autophagy (Basmaciyan et al., 2019).

Atherosclerosis is driven by deposition of LDL in the arterial intima, which triggers endothelial cell activation and inflammation followed by the activation of toll-like receptor (TLR) pathways and the assembly of the NLRP3 inflammasome. Autophagy and cholesterol efflux, which deteriorate with age, are important protective mechanisms against atherosclerosis. Miltefosine activates AMPK and ULK1, inhibits TLR signaling pathway, and reduces NLRP3 inflammasome assembly. In this regard, autophagy enhancement by APCs may have a therapeutic potential in atherosclerosis (Iacano et al., 2019).

Miltefosine enhances autophagy in cutaneous T-cell lymphoma as shown by increased levels of LC3B in MJ (from patients with mycosis fungoides) and Hut78 (from a patient with Sézary syndrome) cells (Yosifov et al., 2014).

#### Perifosine

The second generation APC, perifosine, activates autophagy by inhibiting Akt and the assembly of mTOR raptor and mTOR/rictor complexes and also by increasing degradation of the major components (mTOR, rictor, raptor, p70S6K, and 4E-BP1) in the mTOR axis through an ubiquitin/proteasome-mediated mechanism in human lung cancer cells (Fu et al., 2009). Combination of perifosine with other autophagy modulators may provide benefits in cancer regimens as shown by preclinical studies (Cirstea et al., 2010; Tong et al., 2012). Perifosine-induced autophagy may be protective and induces resistance against its cytotoxic and apoptotic effects which can be restored by addition of an autophagy inhibitor CQ in human chronic myelogenous leukemia cells (Tong et al., 2012). Perifosine can augment the autophagy induced by the mTOR inhibitor sirolimus and cause synergistic cytotoxicity in multiple myeloma cells; this mechanism may present a rationale for designing clinical trials in relapsed/refractory multiple myeloma (Cirstea et al., 2010). Perifosine and also the ether lipid edelfosine can induce autophagy by evoking an ER stress response, as demonstrated by a two- to threefold increased expression of the apoptotic transcription factor CHOP (C/EBP homologous protein)/GADD153 (growth arrest and DNA damage inducible gene 153) in human hepatoblastoma HepG2 cells, but not in human glioblastoma U-87 MG cells. It should be noted that miltefosine was devoid of this ER stress inducing effect (Rios-Marco et al., 2015), but erufosine was able to strongly cause ER stress ((Ansari et al., 2018a) and see below).

A recent study revealed the potential role of APCs against neurodegenerative diseases. Enhancement of autophagy by perifosine may combat against TNF-α induced Akt/mTOR signaling, which promotes microglia polarization toward the neurotoxic M1 phenotype and inflammation (Jin et al., 2018).

#### Erufosine

APC induced autophagy is indicated by increased levels of the autophagic marker LC3B. Kapoor et al. (Kapoor et al., 2012) were the first to demonstrate that erufosine can cause both, apoptosis and autophagy, by affecting the Akt–mTOR signaling pathway (Kapoor et al., 2014). Exposure of oral squamous carcinoma cells to erufosine induced formation of acidic vesicular organelles as tested by acridine orange stain. Erufosine increased the ratio of lipidated LC3B-II to LC3B-I, denoting autophagosome formation in oral squamous carcinoma cells when compared to untreated controls. This effect probably results from the capability of the drug to inhibit the PI3K/Akt/mTOR pathway as a regulator of the autophagy process (Kapoor et al., 2012). The treated tumor cells showed reduced size, detached growth, and membrane blebbing as main characteristics of apoptosis (Kapoor et al., 2012; Kapoor et al., 2014).

In pancreatic ductal adenocarcinoma cells, erufosine showed also a dose-dependent increase in LC3B expression at mRNA and protein levels. Moreover, it caused an increased expression of the autophagy receptor optineurin in Suit2-007 cells (Ali et al., 2019).

Furthermore, erufosine induces endoplasmic reticulum and mitochondrial stress, which is linked to its effects on autophagy, apoptosis, and ROS induction. The association between erufosine and ER stress was shown by silencing and drug-induced inhibition of the ER stress sensors PERK and XBP1, which diminished the cellular effects of erufosine towards e.g. inhibition of proliferation, induction of apoptosis and autophagy (Ansari et al., 2018a).

## Conclusion

In conclusion, autophagy is a multistep process, which can either suppress or favor cancer development. There are four subtypes, which have been named chaperone mediated autophagy, microautophagy, macroautophagy, and selective autophagy. Among other stimuli, autophagy is triggered by the complex 1 of mTOR, which, following its activation, inhibits autophagy induction. Alkylphosphocholine derivatives represent a membrane seeking class of antineoplastic agents that induce autophagy by inhibiting the Akt/mTOR cascade. They primarily interfere with phospholipid turnover and thus modify signaling chains, which start from the cell membrane and modulate PI3K/Akt/mTOR, Ras-Raf-MAPK/ERK and SAPK/JNK pathways. APCs include miltefosine, perifosine and erufosine, which represent the first-, second- and third generations of this class, respectively.

APCs inhibit Akt which acts as the main regulator of cell survival. In human cancers, the autophagic process is usually suppressed *in vitro* and *in vivo* by the constitutively active Akt and its downstream target mTOR. Therefore, exposure to APC derivatives, administered as monotherapy or in combination with other drugs, increases autophagy by reducing Akt/mTOR phosphorylation. Autophagy is a double-edged sword and may result in chemotherapeutic resistance as well as cancer cell death when apoptotic pathways are inactive. APCs display differential autophagy induction capabilities in different cancer cell types. Therefore, autophagy-dependent cellular responses need to be well understood in order to improve the chemotherapeutic outcome.

## Author Contributions

FK, DA, and MB wrote the manuscript. MB composed and edited the review. All authors read and approved the final manuscript.

## Conflict of Interest

The authors declare that the research was conducted in the absence of any commercial or financial relationships that could be construed as a potential conflict of interest.
